# Temporal Pattern of ICAM-I Mediated Regulatory T Cell Recruitment to Sites of Inflammation in Adoptive Transfer Model of Multiple Sclerosis

**DOI:** 10.1371/journal.pone.0015478

**Published:** 2010-11-15

**Authors:** Sebastian Doerck, Kerstin Göbel, Gesa Weise, Tilman Schneider-Hohendorf, Michael Reinhardt, Peter Hauff, Nicholas Schwab, Ralf Linker, Mathias Mäurer, Sven G. Meuth, Heinz Wiendl

**Affiliations:** 1 Department of Neurology, University of Wuerzburg, Wuerzburg, Germany; 2 Department of Neurology – Inflammatory Disorders of the Nervous System and Neurooncology, University of Muenster, Muenster, Germany; 3 Research Laboratories, Schering AG, Berlin, Germany; 4 Department of Neurology, University of Erlangen, Erlangen, Germany; 5 Caritas Hospital Bad Mergentheim, Bad Mergentheim, Germany; 6 Institute of Physiology I – Neuropathophysiology, Muenster, Germany; Julius-Maximilians-Universität Würzburg, Germany

## Abstract

Migration of immune cells to the target organ plays a key role in autoimmune disorders like multiple sclerosis (MS). However, the exact underlying mechanisms of this active process during autoimmune lesion pathogenesis remain elusive. To test if pro-inflammatory and regulatory T cells migrate via a similar molecular mechanism, we analyzed the expression of different adhesion molecules, as well as the composition of infiltrating T cells in an *in vivo* model of MS, adoptive transfer experimental autoimmune encephalomyelitis in rats. We found that the upregulation of ICAM-I and VCAM-I parallels the development of clinical disease onset, but persists on elevated levels also in the phase of clinical remission. However, the composition of infiltrating T cells found in the developing versus resolving lesion phase changed over time, containing increased numbers of regulatory T cells (FoxP3) only in the phase of clinical remission. In order to test the relevance of the expression of cell adhesion molecules, animals were treated with purified antibodies to ICAM-I and VCAM-I either in the phase of active disease or in early remission. Treatment with a blocking ICAM-I antibody in the phase of disease progression led to a milder disease course. However, administration during early clinical remission aggravates clinical symptoms. Treatment with anti-VCAM-I at different timepoints had no significant effect on the disease course. In summary, our results indicate that adhesion molecules are not only important for capture and migration of pro-inflammatory T cells into the central nervous system, but also permit access of anti-inflammatory cells, such as regulatory T cells. Therefore it is likely to assume that intervention at the blood brain barrier is time dependent and could result in different therapeutic outcomes depending on the phase of CNS lesion development.

## Introduction

Thymus-derived (naturally occurring) regulatory T cells (T_reg_) are essential for regulating peripheral autoimmune tolerance and thereby inflammation in the context of infection, autoimmunity and transplant rejection [1]. It has been demonstrated that antigen-specific T_reg_ have the capacity of limiting autoimmune tissue damage in disease models for multiple sclerosis, rheumatoid arthritis and type I diabetes [2], [3], [4], [5], [6], [7].

*In vitro*, natural T_reg_ are hypoproliferative and suppress T effector cell (T_eff_) under autologous and alloreactive coculture conditions [8], [9]. Conceptually T_reg_ influence physiological and pathological immune reactions at different levels, thereby influencing parenchymal immune homeostasis. One concept assumes that T_reg_ are equipped with a higher propensity to migrate in order to prevent T_eff_ at target sites of emerging inflammation [10]. However, the origin and temporal pattern of T_reg_ actions in acute and chronic autoimmune tissue inflammation *in vivo* is still largely elusive.

It has been proposed that in a model of MS, experimental autoimmune encephalomyelitis (EAE) T_reg_ are unable to reach the central nervous system (CNS), but prevent migration of autoreactive T_eff_ into the target organ [3]. More recent reports, however, demonstrate that T_reg_ accumulate within the murine CNS during EAE [4] and limit EAE relapses in the CNS [11], [12]. However, the underlying mechanism of T_reg_ migration to the target organ during CNS lesion development remains largely unknown.

In general, cell trafficking to the CNS is a highly regulated process and involves different components on both interacting compartments – T cells and the blood brain barrier (BBB) [13], [14]. Under physiological conditions, highly specialized brain endothelial cells are key components that limit trans- and paracellular movement of molecules and cells [13]. Under inflammatory conditions, however, structural integrity of the BBB seems to collapse and transendothelial trafficking increases [13], [14], [15]. The involvement of various chemokines, as well as the expression of cellular adhesion molecules and tight junction proteins has been described [14]. Thereby, especially the interaction between the alpha4beta1 integrin very late antigen (VLA)-4 and leukocyte function associated antigen (LFA)-1 with their respective immunoglobulin-like ligands vascular cell adhesion molecule (VCAM)-I and intercellular adhesion molecule (ICAM)-I were shown to be essential for leukocyte adhesion and migration into the CNS *in vivo* [14], [16], [17], [18]. Similar mechanisms were described to play a role for the migration of T_eff_ in EAE [17], [19], [20], [21].

To test if these molecular mechanisms are also involved in the regulation of T_reg_ migration in an *in vivo* model of autoimmune inflammation, we performed adoptive transfer (AT)-EAE in rats. This model is considered to be in particular useful to evaluate the infiltration of leukocytes into the CNS [22], [23] and opens up a rational to define molecular target structures for therapeutical intervention.

## Materials and Methods

### Animal experiments

All animal experiments were approved and conducted in accordance with the laws and regulations of the regulatory authorities for animal care and use in Lower Franconia, Germany (ID 55.2-2531.01-75/07). 6–8 week old female Lewis rats with body weights ranging from 140–160 g were purchased from Harlan (Harlan Winkelmann, Borchen, Germany). AT-EAE was induced by intravenous injection of freshly activated myelin basic protein (MBP)-specific T cell blasts, using a dose of 8×10^6^ T cells respectively to generate EAE. Animals were weighed and their disease state was scored by two blinded examiner (SD, SGM) according to clinical signs. This score ranged from 0 to 10; scores were as follows: 0 = normal; 1 = limp tail, impaired righting; 2 = gait ataxia; 3 = moderate paraparesis; 4 = tetraparesis; 5 = death.

For blocking experiments animals were injected i.p. with 1 mg of a monoclonal murine antibody against rat-ICAM-I (clone 1A-29, BD Biosciences, Heidelberg, Germany) and 1.5 mg of a monoclonal antibody against rat-VCAM-I (clone MR106, eBioscience, San Diego, CA) in a volume of 1 ml PBS i.p. Sham-treated animals received 1 ml PBS only, EAE controls were untreated.

### Preparation of microparticles

Poly(butyl-2-cyanoacrylate) (PBCA) stabilized air-filled microparticles were generated and used as described earlier [24], [25]. The target-specificity of streptavidin loaded microparticles was generated immediately prior to their use by adding either 50 µg biotinylated anti-rat-ICAM-I- (clone 1A-29, BD Biosciences) or anti-rat-VCAM-I- (clone MR106, eBioscience) antibodies to a suspension containing 5×10^8^ microparticles (10 min incubation time).

### Immunocytochemistry

Native brains and spinals cord were prepared at the respective time points and snap-frozen at −70°C. Immunohistochemistry was performed on 10 µm cryosections according to standard protocols with a mouse anti-rat-ICAM-I- antibody (clone 1A-29, BD Biosciences, 1∶100), anti-rat-VCAM-I- antibody (clone MR106, eBiosciences, 1∶100), mouse anti-rat-CD4- (clone 15-8A2, Holland Biotechnology bv, Rotterdam, Netherlands, 1∶8000), mouse anti-rat-CD8- (clone R1-10B5, Seikagaku Kogyo co., Tokyo, Japan, 1∶100), and anti-rat-FoxP3 (clone 150D, biolegend, SanDiego, CA, 1∶500). Specificity of staining was confirmed by omitting the primary antibody as a negative control. After blocking endogenous peroxidase activity with 3% H2O2 and 0.2 M sodium azide in methanol, primary antibodies were detected using the ABC system (DAKO, Hamburg, Germany) with 3,3′-diaminobenzidine tetrahydrochloride as substrate. All sections were counterstained with haematoxylin for 30 s, dehydrated and mounted in Vitro-clud® (R. Langenbrinck, Emmendingen, Germany). Quantitative analysis of inflammatory infiltrates within the spinal cord was performed as previously described [26]. Briefly, an average of 10 spinal cord cross-sections (cervical, thoracic and lumbal, respectively) from 4–8 rats per timepoint were used for evaluation. Complete sections were counted by blinded observers and cells were calculated as cells per slide.

### Flow cytometry

Quantitative analysis of CNS infiltrates during AT-EAE by flow cytometry was done during disease progression, at disease maximum and during early remission using a method described by Magnus et al., 2005 [27]. Briefly, rats were sacrificed with CO_2_ and spinal cords were flushed out of the spinal column with sterile PBS, the brain was prepared and used in toto. CNS tissue was homogenized and strained through a 70 µm nylon filter (BD Biosciences). After centrifugation, the cell pellet was resuspended in 9 ml Percoll and mixed with 20 ml PBS. The gradient was centrifuged at 600 g for 25 min at room temperature (21 to 23°C). The interphase cells were collected and washed before staining. Spleens were washed in PBS and homogenized by pressing through a 40 µm cell strainer. After washing, splenocytes were separated by gradient centrifugation. Cells were counted and diluted to a concentration of 20×10^6^ splenocytes/ml in FACS buffer (made from PBS, 1% FCS and 0.1% sodium acide). For flow cytometry, cells were stained with the following directly labelled antibodies for 30 min at 4°C: CD3, CD4, CD8, CD25 and CD28 and their respective isotype antibodies (all BD Biosciences). FoxP3 staining was performed according to the manufacturer's instructions for intracellular staining using a FoxP3 anti-rat staining kit (clone: FJK-16s; eBioscience). Specific staining was assessed by applying accordant isotype control antibodies. Cells were washed and analyzed using a FACSCalibur (FACS calibur, BD Biosciences) with CellQuest software.

### Statistical analysis

The experimental results are expressed as means +/− standard deviation. Significant differences between experimental groups were analysed by using a t-test modified for small samples for parametric data or Mann-Whitney U test for non-parametric datasets. A *p* value of <0.05 was considered statistically significant. The statistical Software used was Statistica 3.0 (Statsoft, Tulsa, OK, USA).

## Results

### Cellular composition of inflammatory CNS lesions over time: evidence for the presence of CD4CD25FoxP3 regulatory cells in the remission phase of the disease

**Figure 1:**
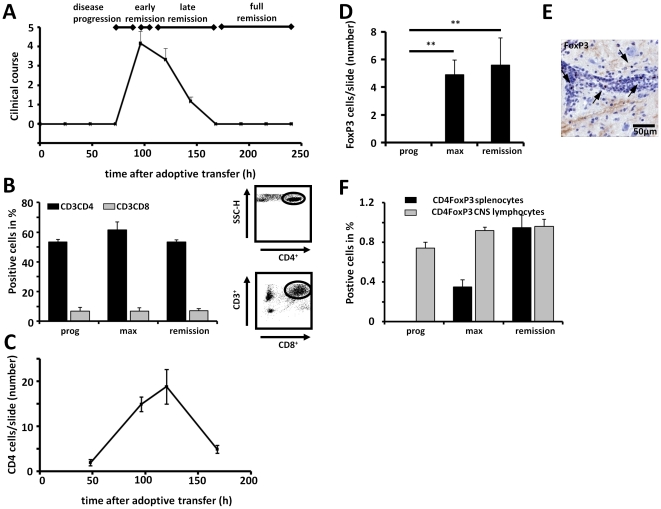
Migration of T_eff_ and T_reg_ in AT-EAE. (A) Clinical course of AT-EAE (n = 8). (B) CD4 T cells are the predominant cell type within the CNS. Both numbers of CD3CD4 (black bars) and CD3CD8 T cells (grey bars) are slightly increasing after 96 hours at the disease maximum (max) compared with the progression stage (prog), but show persistence in the CNS when clinical symptoms are already remitting (early remission). (C) Quantitative analysis of CD4 positive profiles in frozen spinal cord sections shows an increase of CD4 infiltrates to the time point of disease maximum. Persistence of CD4 cells in early clinical remission (120 h) is also found on the histological level. (D) Quantification of FoxP3 immunoreactive cells in the spinal cord of EAE animals were almost absent during disease progression (prog), but show a significant increase during the clinical maximum (max) and were even more prominent in the stages of early clinical remission. (E) Representative immunostaining of lumbar spinal cord cryosections with antibodies to FoxP3 demonstrates the presence of FoxP3 immunoreactive cells in EAE lesions (arrows). (F) Quantification of CD4FoxP3 positive cells in the CNS over time by flow cytometry. CD4FoxP3 positive cells (grey bars) show an increase in early clinical remission compared to the clinical maximum and the progression state where FoxP3 T_reg_ could not be detected at all. CD4FoxP3 splenocytes (black bars) served as positive control. Values represent clinical score means ± SEM. ** p<0.01, * p<0.05

AT-EAE induced animals showed the first clinical symptoms ∼72 hours after cell transfer. Disease progression thereafter is rapid, leading to the peak of the disease ∼96 h post cell injection (**Figure 1A**). Improvement of clinical signs began 96–110 hours after cell transfer (in the following also named early remission). Usually the recovery phase lasted 48–72 hours (in the following named late remission), approximately 240 hours after cell transfer animals showed completely recovery (full remission; **Figure 1A**).

In a first step, we analyzed the cellular composition of the inflammatory CNS lesions in AT-EAE at different time points. Quantitative analysis of infiltrating immune cells by flow cytometry analysis from homogenized CNS (brain and spinal cord; **Figure 1B**) displayed high amounts of CD4 T cells within the CNS of AT-EAE animals at the disease maximum. Interestingly, the CD4 T cell population persisted also in the stages of early clinical remission (**Figure 1B**). A percentage of approximately 10% CD8 T cells could be observed throughout the disease course (despite the fact that pure CD4-MBP-specific blasts have been injected into mice) (**Figure 1B**). These results could be confirmed using immunohistochemistry (**Figure 1C**). In contrast, at disease maximum and even more pronounced in stages of early clinical remission a significant increase of FoxP3+ expressing T_reg_ was notable (**Figure 1D, E**). Performing immunohistochemistry, T_reg_ could hardly be detected on serial sections during the phase of disease progression (data not shown). To confirm these histopathological findings, flow cytometry analysis from homogenized CNS (brain and spinal cord) was performed (**Figure 1F**). As a positive control, T_reg_ were also identified within the spleen of AT-EAE animals. The rates of T_reg_ within the spleen were comparable to the numbers known from the literature [28]. Flow cytometry analysis data corroborated immunohistochemistry showing virtually no FoxP3 cells during disease progression in the CNS of the animals, but a constant increase of regulatory cells from disease maximum to stages of early clinical remission (**Figure 1F**) indicating their important role in regulating the resolution phase of the CNS immune responses.

### Time course of adhesion molecule expression in EAE: persisting expression of ICAM-I and VCAM-I during disease remission

**Figure 2:**
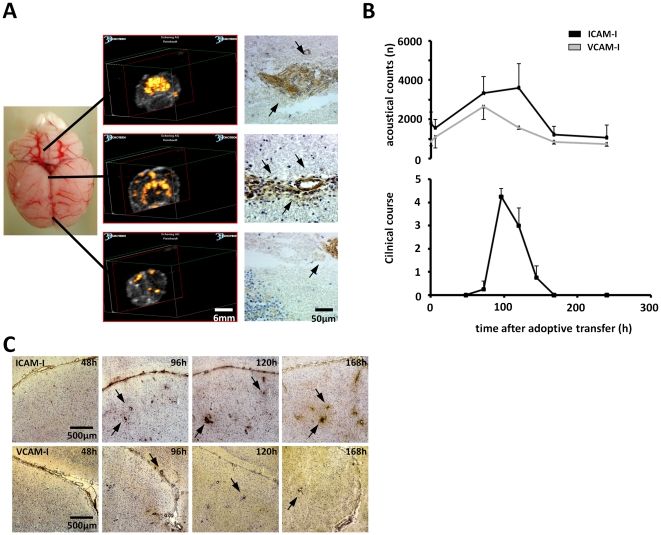
Imaging of ICAM-I and VCAM-I temporal expression pattern during AT-EAE with the SPAQ technology. (A) The left panel shows an AT-EAE rat brain with corresponding images of the brainstem, midbrain and frontal cortex. The yellow spots represent the SAE effect that is generated by ICAM-I specific microparticles. The SAE signal is predominantly derived from the cerebellum/brainstem and the periventricular region. Corresponding histological sections are shown. A strong vascular ICAM-I expression (arrows) can be observed. (B) Ultrasound derived sequential quantification of ICAM-I and VCAM-I expression in AT-EAE shown as acoustical counts in relation to the clinical course. Both ICAM-I and VCAM-I are upregulated in parallel to the clinical disease course and show a delayed return to baseline level in the clinical remission. (C) Serial immunohistochemistry stainings with antibodies to ICAM-I and VCAM-I of periventricular brain cryosections 48 h (progression), 96 h (maximum), 120 h (early remission) and 168 h (late remission) after induction of EAE. Arrowheads demonstrate the ICAM-I and VCAM-I immunostaining at the cerebral vessels. Representative examples are shown. Scale bar represents 500 µm. Values represent clinical score means ± SD. *p<0.05.

In a next step we investigated some main components involved in the regulation of leukocyte trafficking. Therefore we analyzed the expression of the adhesion molecules ICAM-I and VCAM-I at baseline (0 h) and 6 h, 72 h, 120 h, 168 h and 240 h after cell transfer by using ICAM-I and VCAM-I specific microparticles for ultrasound-based molecular imaging allowing serial analysis. These microparticles mark ICAM-I and VCAM-I at the BBB with the stimulated acoustic emission (SAE) effect, generating a characteristic imaging pattern with a strong periventricular signal, as well as a strong signal of the cerebellar and brain stem region (**Figure 2A**).

For *in vivo* imaging of living anesthetized animals, the periventricular signal was quantified by using the SPAQ technology and the signal strength that correlates with the amount of adhesion molecules, was expressed as acoustical counts (AC).

Within the course of AT-EAE, the molecular expression of ICAM-I and VCAM-I changed parallel to the development of clinical signs and symptoms (**Figure 2B**). Interestingly, in the phase of clinical remission, expression levels only of VCAM-I showed a slow decline to baseline similar to the clinical course, whereas ICAM-I stayed up-regulated also during the stages of early clinical remission (n = 5; p = 0.0415). After ∼240 h expression pattern of both adhesion molecules returned to baseline levels.

In order to confirm our kinetic *in vivo* imaging ultrasound results, we performed an immunocytochemical analysis of ICAM-I and VCAM-I expression within the brain of AT-EAE animals at different time points (**Figure 2C**). In the preclinical stage, ICAM-I and VCAM-I could be detected, but the expression was low in comparison to the expression pattern detected at disease maximum (96 h). In accordance with our imaging results, the strong expression pattern for ICAM-I persisted also in the stages of early clinical remission (120 h). A reduction of the perivascular ICAM-I and VCAM-I staining was only seen in the stages of late clinical remission (168 h) when the animals already showed full recovery (**Figure 2C**).

### Time dependence of adhesion molecule blockade with antibodies to ICAM-I

As adhesion molecule expression stayed elevated during early clinical remission, we analyzed if the constant up-regulation of adhesion molecules in AT-EAE can not only influence the migratory capacity of T_eff_, but also represent a relevant mechanism to allow T_reg_ to enter the CNS. To elucidate the possible impact and time-dependence of cell adhesion molecule expression at the BBB, we performed *in vivo* inhibition experiments.

**Figure 3:**
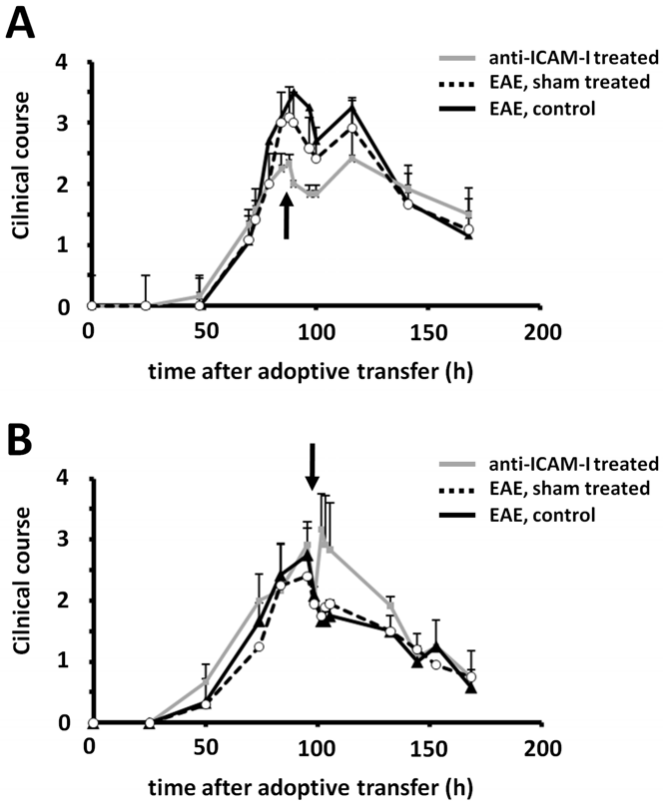
Time dependency of ICAM-I blockade at the BBB with monoclonal antibodies in AT-EAE. Clinical courses of anti-ICAM-I treated, sham treated and non treated animals are shown. Time points of injection are marked with an arrow. (A) I.v. administration of 1 mg anti-ICAM-I monoclonal antibodies in the clinical progression phase (80 h after induction) results in a significant reduction of disease severity in mAB treated animals compared to PBS treated and non treated control animals. (B) Blocking of ICAM-I by mAB in the early remission phase (105 h after induction) leads to a significant increase in disease severity in mAB treated animals in comparison to PBS treated and non treated animals, respectively. n = 8 per group. Values represent clinical score means ± SD. *p<0.05.

Therefore, AT-EAE animals were treated with a high dose of purified monoclonal antibodies to ICAM-I, either in the phase of disease progression or in early clinical remission. The administration of 1 mg anti-ICAM-I antibody during the phase of disease progression resulted in a significant suppression of EAE in comparison to sham-treated animals (**Figure 3A**). In contrast however, the administration of anti-ICAM-I in the phase of early clinical remission resulted in a short but notable aggravation of clinical symptoms (**Figure 3B**). These data suggest that prevention of ICAM-I-mediated T-cell CNS entry has phase dependent consequences for the development or resolution of CNS lesions. Aggravation of clinical symptoms after ICAM-blockade in the early remission phase correlated with the phase of FoxP3 T_reg_ entry into the CNS.

Of note, using a blocking anti-VCAM-I antibody both during the phase of disease progression and during early clinical remission showed no significant effect on the clinical course of AT-EAE (**Figure S1**) and no marked changes in whole brain FACS analysis of CD4 and FoxP3 cell populations.

Taken together, our blocking experiments indicate an important role for ICAM-I in terms of CNS trafficking of both inflammatory T_eff_ cells and T_reg_.

## Discussion

To test if pro-inflammatory and regulatory T cells migrate via a similar molecular mechanism, we analyzed the expression pattern of ICAM-I and VCAM-I, as well as the composition of infiltrating T cells in AT-EAE in rats. Whereas VCAM-I parallels the clinical course, ICAM-I remained upregulated in the phase of early clinical remission. However, the composition of infiltrating T cells found in the developing versus resolving lesion phase changed over time, containing increased numbers of T_reg_ in the phase of clinical remission.

In order to test the relevance of the expression of cell adhesion molecules, animals were treated with purified antibodies to ICAM-I and VCAM-I either in the phase of active disease or in early remission. While treatment with a blocking ICAM-I antibody in the phase of disease progression led to a milder disease course, administration during early clinical remission aggravates clinical symptoms. Treatment with anti-VCAM-I at different timepoints had no significant effect on the disease course.

During inflammatory diseases of the CNS such as MS or its prototype animal model EAE, the BBB has an active role in regulating cell entry into the target organ and is characterized by enhanced expression of traffic signals like adhesion molecules and chemokines [14]. If distinct mechanisms play a role for specific T cell subsets is not known so far. Thus, knowledge on the molecular mechanisms involved in cell-type specific migration across the BBB is not only important for our understanding of the pathogenesis of autoimmune inflammation of the CNS, but can also be relevant for the development or understanding of treatments acting at the BBB.

Intensive research during the last decade has pointed to a unique interaction of activated T cells with the BBB. By using intravital fluorescence videomicroscopy in the EAE model, it was shown that initial capture and subsequent firm adhesion of activated T cells to the brain endothelium is mediated by an interaction between VLA-4 and VCAM-I [21] while the interaction between LFA-1 and its ligand ICAM-I on brain endothelial cells is important for transmigration of activated cells into the CNS [19]. Although these results have clarified the distinct molecular interactions at the BBB, little is known about the temporal regulation pattern of these important cell adhesion molecules during the entire course of AT-EAE. We here used our newly developed ultrasound-based molecular imaging and quantification method SPAQ [24], [25] addressing this important question of temporal ICAM-I and VCAM-I expression in a serial manner. These data were corroborated with more conventional assessments of animal groups assessed by immunohistochemistry and CNS flow cytometry analysis. These data showed up-regulation of ICAM-I and VCAM-I that already preceded the development of clinical symptoms and paralleled the development of clinical disease. However, only VCAM-I slowly returned to its baseline parallel to clinical recovery, whereas ICAM-I showed an increase during the stages of early clinical remission.

This suggested that BBB activation and cellular transmigration is necessary also for immune cells actively participating in counterbalancing or terminating proinflammatory T_eff_ responses. By analysing the cellular composition of the inflammatory CNS lesions, we discovered that T_reg_ become detectable in the respective stage of early clinical remission, while they are absent in the progression phase or under normal conditions, indicating that persisting adhesion molecule expression might also allow transmigration of these suppressive immune cell components. This regulatory cell population has the potential to directly or indirectly alter the activation and differentiation of pathogenic T cells [29]. There is evidence that T_reg_ in the CNS provide beneficial effects in neuroinflammation and the contribution of T_reg_ in recovery and protection from autoimmune encephalomyelitis has been demonstrated [7], [30]. There is also accumulating data in humans to support the assumption that regulatory T cell populations specifically migrate or accumulate in the CNS compartment, in order to combat inflammation [31], [32], [33]. However, there is still some controversial debate where exactly T_reg_ modulate the inflammatory response. Using the monoclonal anti-CD28 antibody JJ316 that induces T_reg_, Tischner et al. argue that T_reg_ cell action on T_eff_ is mainly confined to the secondary lymphoid organs. However, the authors did find T_reg_ within the spinal cord on day six after disease induction and suggested that T_reg_ might also play an additional role at sites of inflammation, e.g. in the resolution of ongoing autoimmune diseases or in preventing relapses [34].

As a proof of concept we here provide experiments in which we applied high doses of a blocking anti-ICAM-I monoclonal antibody at different time points. While an early administration of the antibody during the stage of disease progression resulted in a significant amelioration of the clinical disease, a subsequent application of these blocking antibodies in the stage of early clinical remission resulted in a clinical deterioration of the treated animals. As our first analyses show that in our model T_reg_ migrate to the CNS to a later timepoint, it can be assumed that this short term aggravation of the clinical symptoms might be due to the prevention of counterregulatory or protective immune cells recruited into the CNS, such as T_reg_. This result is very much compatible with a concept assuming that T_reg_ also act at the blood brain barrier or within the parenchyma to counterbalance inflammation.

Although our experiments thus far do not present direct experimental evidence, it is suggestive that interaction of LFA-1/ICAM-I but not VLA-4/VCAM-I is necessary for T_reg_ to enter the CNS, at least under the given experimental conditions. This assumption is corroborated by recent findings that T_reg_ express comparable levels of LFA-1 und VLA-4 as T_eff_ [35]. It is tempting to speculate that molecular interactions between pro-inflammatory and T_reg_ and the BBB, namely that both pro-inflammatory and T_reg_ use ICAM-I for transmigration into the CNS. However, we suppose that these interactions between different T cell subsets and the BBB occur at different time points in the course of EAE.

According to these observations we assume that the outcome of therapeutic interventions at the BBB is time dependent. Our hypothesis might be an explanation of the conflicting results published on the treatment of EAE with anti-ICAM-I antibodies. While some studies including a study from the Wuerzburg group could show a clear beneficial treatment effect [36], [37]. Others however could not reproduce this positive effect but show rather a worsening of the disease [38], [39].

Our data also have important implications for understanding current therapeutic approaches acting at the BBB. There is no doubt that we hold powerful drugs that can block transmigration of inflammatory cells into the CNS to a great extent [40], [41]. However, the timepoint of treatment seems to be important, as unselective blockade of adhesion molecules at the BBB, not only reduce trafficking of encephalitogenic T_eff_, but also avoid the migration of T_reg_ that could limit CNS lesion development or be required for CNS immune surveillance.

In summary, our results indicate that adhesion molecules are not only important for capture and migration of pro-inflammatory T cells into the central nervous system, but also permit access of anti-inflammatory cells, such as regulatory T cells. Therefore it is likely to assume that intervention at the blood brain barrier is time dependent and could result in different therapeutic outcomes depending on the phase of CNS lesion development.

## Supporting Information

Figure S1 **No influence of VCAM-I inhibition on the clinical course of AT-EAE.** Clinical courses of anti-VCAM-I treated (early and late intervention) and non treated animals are shown. Phases of treatment are indicated in green (early intervention) and red (late intervention). I.v. administration of 1.5 mg/kg anti-VCAM-I monoclonal antibodies in the clinical progression phase (2d after induction) as well as in the early remission phase (105 h after induction) has no influence on the disease severity. N = 8 per group. Values represent clinical score means ± SD. (TIF)
