# Supplementary figures and images for: Temporal Pattern of ICAM-I Mediated Regulatory T Cell Recruitment to Sites of Inflammation in Adoptive Transfer Model of Multiple Sclerosis
Source: PLoS One. 2010 Nov 15;5(11):e15478. doi: 10.1371/journal.pone.0015478 (PMC2981557; doi:10.1371/journal.pone.0015478)

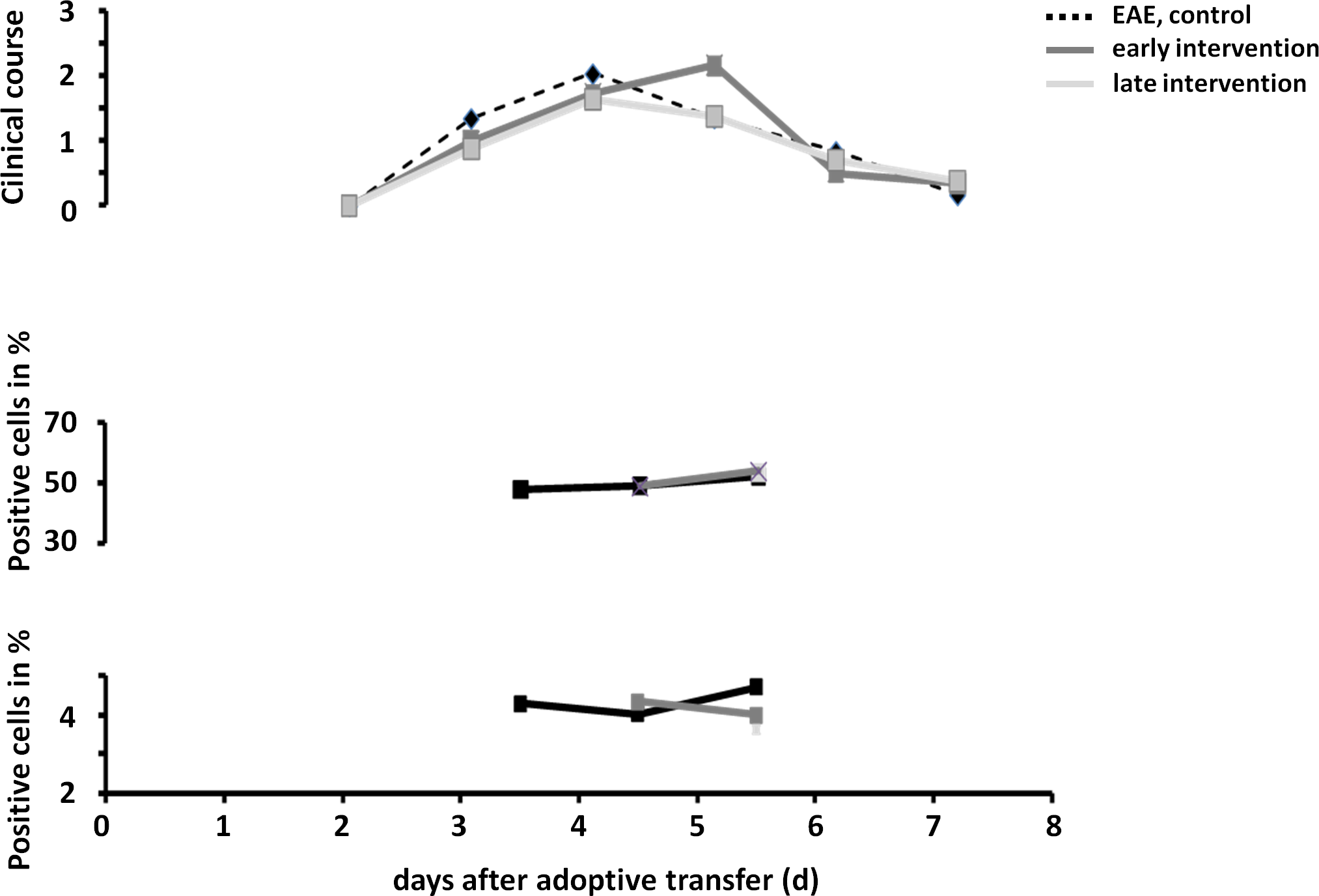

Supplement: Figure S1 — No influence of VCAM-I inhibition on the clinical course of AT-EAE. Clinical courses of anti-VCAM-I treated (early and late intervention) and non treated animals are shown. Phases of treatment are indicated in green (early intervention) and red (late intervention). I.v. administration of 1.5 mg/kg anti-VCAM-I monoclonal antibodies in the clinical progression phase (2d after induction) as well as in the early remission phase (105 h after induction) has no influence on the disease severity. N = 8 per group. Values represent clinical score means ± SD. (TIF) [file pone.0015478.s001.tif]
